# Excessive weight loss in exclusively breastfed full-term newborns in a Baby-Friendly Hospital

**DOI:** 10.1016/j.rppede.2016.03.003

**Published:** 2016

**Authors:** Maria Aparecida Mezzacappa, Bruna Gil Ferreira

**Affiliations:** a Faculdade de Ciências, Universidade Estadual de Campinas (Unicamp), Campinas, SP, Brazil

**Keywords:** Newborn, Breastfeeding, Weight loss, Cesarean section

## Abstract

**Objective:**

To determine the risk factors for weight loss over 8% in full-term newborns at postpartum discharge from a Baby Friendly Hospital.

**Methods:**

The cases were selected from a cohort of infants belonging to a previous study. Healthy full-term newborns with birth weight ≥2.000g, who were exclusively breastfed were included and excluded twins and those undergoing phototherapy as well as those discharged after 96h of life. The analyzed maternal and neonatal variables were maternal age, parity, ethnicity, type of delivery, maternal diabetes, gender, gestational age and appropriate weight for age. Adjusted multiple and univariate Cox regression analyses were used, considering as significant *p*<0.05.

**Results:**

We studied 414 newborns, of whom 107 (25.8%) had excessive weight loss. Through the univariate regression, risk factors associated with weight loss>8% were cesarean delivery and older maternal age. At the adjusted multiple regression analysis, the model to explain the weight loss was cesarean delivery (Relative risk 2.27, 95% of Confidence Interval 1.54–3.35).

**Conclusions:**

The independent predictor for weight loss>8% in exclusively breastfed full-term newborns in a Baby-Friendly Hospital was the cesarean delivery. It is possible to reduce the number of cesarean sections to minimize neonatal excessive weight loss and the resulting use of infant formula during the first week of life.

## Resumo

**Objetivo:**

Determinar os fatores de risco para perda de peso acima de 8% em recém-nascido a termo por ocasião da alta pós-parto de um Hospital Amigo da Criança.

**Métodos:**

Os casos foram selecionados de uma coorte de recém-nascido, pertencentes a um estudo prévio. Foram incluídos recém-nascidos a termo com peso ao nascer ≥2.000g, saudáveis e amamentados exclusivamente, excluídos os gemelares, os recém-nascidos que usaram fototerapia e aqueles com alta hospitalar após 96 horas de vida. As variáveis maternas e neonatais estudadas foram idade materna, paridade, raça, tipo de parto, diabete materna, sexo, idade gestacional e adequação do peso para idade. Foram usadas as análises de regressão de Cox univariada e múltipla ajustadas e foi considerado significativo *p*<0,05.

**Resultados:**

Foram estudados 414 recém-nascidos dos quais 107 (25,8%) tiveram perda excessiva de peso. Pela regressão univariada, os fatores de risco associados à perda de peso >8% foram parto cesárea e maior idade materna. Pela análise de regressão múltipla ajustada, o modelo para explicar a perda de peso foi o parto cesárea (Risco Relativo: 2,27 e Intervalo de Confiança 95%: 1,54-3,35).

**Conclusões:**

O preditor independente para perda de peso maior do que 8% em recém-nascidos a termo amamentados exclusivamente em um Hospital Amigo da Criança foi a cesárea. É possível que a redução do número de cesáreas possa minimizar a perda de peso neonatal excessiva e o consequente uso de fórmula láctea na primeira semana de vida.

## Introduction

Almost all newborns lose weight on the first days of life.^1^ Given this high frequency, the authors call it physiological weight loss.^2^ Most studies suggest that the loss corresponds mainly to fluid reduction,^1^ but it is also a consequence of the use of adipose tissue as a source of energy by the newborns.^2^

On the first 2-3 days of life,^3^ newborns that are exclusively breastfed lose on average between 5% and 7% of their birth weight.^1^ The maximum physiological limits of weight loss for newborns that are exclusively breastfed are controversial. Thus, a weight loss of 10% can be considered normal or acceptable,^4^^-^ 6 although there have been references about 7% values.^7^

The evolution of the newborn's weight on the first days of life is used as an indicator of breastfeeding adequacy.^7^ Thus, the percentage of weight reduction in relation to birth weight can be one of the parameters used for the introduction of formula.^8^

The subject has attracted increasing interest due to the large number of aspects with low levels of evidence. However, the percentage of weight loss that indicates formula supplementation, the decrease in weight compatible with safe hospital discharge and the time required for weight recovery remain to be defined.^9^

UNICEF's Baby Friendly Hospital initiative^10^ recommends exclusive breastfeeding, but the short-term impact of this practice on weight evolution is little known.

Regarding the possible markers associated with weight loss, there are publications involving infants with partial feeding, i.e., babies that are breastfed and also receive formula supplementation.^11^^,^ 12 In these studies, the factors associated with weight loss are multiple and among them is the cesarean delivery. On the other hand, there have been few studies in newborns that are exclusively breastfed^3^^,^ 13 and in Baby-Friendly Hospitals.^14^^,^ 15 The aim of this study was to determine the risk factors for weight loss greater than 8% in full-term newborns that are exclusively breastfed in a Baby-Friendly Hospital.

## Method

A secondary analysis was performed on data from a previously published study (*n*=608) carried out from 06/2008 to 10/2008.^16^ The weight gain of a cohort of full-term newborns with birth weight ≥2.000g and ≥37 weeks gestational age was prospectively assessed at birth and at hospital discharge. Newborns that received formula supplementation or exclusive formula, twins, newborns whose discharge occurred after 96h of life and those submitted to phototherapy during hospitalization after birth were excluded.

The newborns were weighed at birth and on the day of discharge, without clothes, using a Filizola™ scale, with a sensitivity of 5g. The study site is a public, tertiary hospital, which has adhered to the 10 recommended steps and received the title of a Baby-Friendly Hospital 12 years ago.^10^

Hospital discharge at this service routinely occurred after 48h of life in cases of vaginal delivery and 72h after cesarean delivery. The mean length of stay in this cohort of newborns was 58.9±9.9h, with a minimum of 41 and maximum of 96h, according to the exclusion criterion.

The following maternal independent variables were assessed: age, ethnicity, parity, type of delivery and history of diabetes, based on the results of the glucose curve during the prenatal period. Among the neonatal variables, the following were assessed: gender, birth weight, adequate weight for age, weight at discharge and hospital length of stay. The weight at discharge was obtained in the morning of the discharge day. Gestational age^17^ was established in the delivery room and the adequacy of weight for gestational age was defined according to the intrauterine growth curve of Alexander et al.,^18^ using the birth weight. Weight loss in percentage, at the time of hospital discharge, was considered as the percentage difference between the birth weight and the weight measured at discharge.

The dependent variable was excessive weight loss, considered when there was a reduction>8% in weight at the hospital discharge in relation to the birth weight.

The information on the variables was added to the previous study database^16^: adequacy of weight for age and history of maternal diabetes. Data analysis was performed using the SAS System for Windows version 9.1.3. Statistical analysis was performed using chi-square test, Student's *t*-test, Kruskal-Wallis test, univariate and multiple Cox regression, adjusted by the time of discharge, as newborns with vaginal and cesarean section delivery had different hospital length of stay. The variable selection process used in the regression analysis was the stepwise, in which, at each step, all combinations are tested. All variables were entered into the model, regardless of the *p*-value in the univariate analysis. Relative risk (RR) values and 95% confidence interval (95%) were established and values were considered significant for *p*<0.05.

The Institutional Review Board of the institution approved the original project and waived consent for this secondary analysis study.

## Results

**Figure 1:**
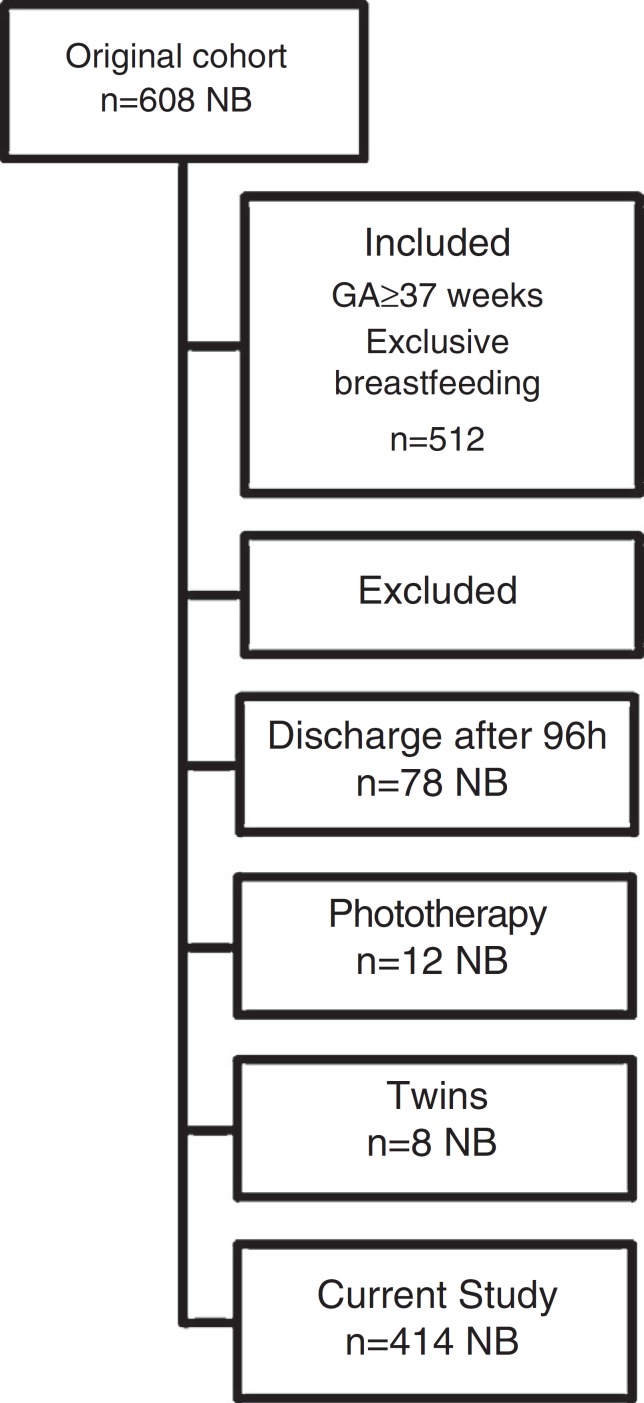
Cohort composition based on the original study.

The study included 414 newborns, according to the criteria of inclusion and exclusion explained in the Method's section and in Fig. 1. In relation to birth weight, the mean±SD was 3.319±409g, the median was 3.305g and the interquartile range was 3.005-3.595g. The mean weight loss in this sample was 6.4±2.5%. Of the total, 107 (25.8%) showed excessive weight loss, with a mean loss of 9.4%±1.1% (range: 8.1-13.6%). In 20 newborns (4.8%) weight loss at discharge was higher than 10%. Hospital length of stay was significantly different between the groups with and without excessive weight loss; hospital discharge occurred, respectively, after 61.4±9.9h vs. 58.0±9.8h (*p*=0.003). Table 1 shows the distribution of mean weight loss and the frequency of weight loss>8%, according to the gestational age. Weight loss did not differ between the gestational ages. The frequency of cesarean section was significantly different (*p*<0.001) among the gestational ages (Table 1).

**Table 1:** Mean±SD and median weight loss values at hospital discharge and the relative frequency of cesarean delivery and weight loss over 8%, according to gestational age (*n*=414).

GA	*n*	Cesarean %^a^	Mean±SD	Median^b^	Loss>8% (%)
37	50	56.2	6.9±2.8	7.2	34.0
38	59	18.0	6.1±2.4	6.5	22.0
39	104	44.4	5.9±2.5	6.1	20.2
40	141	54.9	6.7±2.4	6.8	30.5
41	60	46.3	5.8±2.4	6.4	21.7

GA, gestational age; *n*, number of cases; %, percentage; SD, standard deviation.

a *p*<0.001 at the chi-square test.

b *p*=0.053 at the Kruskal-Wallis test at the comparison of the median weight loss between the gestational ages.

Table 2 shows the risk factors at the univariate regression. Risk factors associated with weight loss>8% were cesarean delivery and older maternal age (Table 2). The model obtained by multiple regression analysis adjusted for the time of discharge that offered the best explanation for weight loss in this sample corresponded to cesarean section, which independently increased the risk of loss>8% by 2.27-fold (RR 2.27, 95%CI 1.54-3.35; *p*<0.0001).

**Table 2:** Risk factors for weight loss>8% at hospital discharge in full-term newborns receiving exclusive breastfeeding, according to the adjusted univariate logistic regression analysis (*n*=414).

	>8% *n*=107	≤8% *n*=307	*p*-value^a^	RR^b^	95%CI^b^
Maternal age (years; mean±SD)	26.4±6.2	24.6±6.3	0.034	1.03	1.00-1.06
Parity (mean±SD)	0.8±0.9	0.9±1.2	0.335	0.91	0.76-1.09
Black ethnicity,^c^ *n* (%)	36 (33.6)	124 (40.3)	0.163	0.75	0.50-1.12
Maternal diabetes, *n* (%)	4 (3.7)	4 (1.3)	0.263	1.77	0.65-4.84
Cesarean delivery, *n* (%)	54 (50.4)	74 (24.1)	<0.0001	2.16	1.47-3.18
Female gender, *n* (%)	56 (52.3)	144 (46.9)	0.397	1.17	0.80-1.72
GA 37 weeks,^d^ *n* (%)	17 (15.8)	33 (10.7)	0.488	1.12	0.65-1.94
Birth weight (g; mean±SD)	3337±415	3313±407	0.778	1.00	1.00-1.00
AGA,^e^ *n* (%)	89 (83.1)	258 (84.0)	0.375	0.77	0.38-1.53

SD, standard deviation; g, grams; *n*, number of cases; GA, gestational age; AGA, appropriate for gestational age.

a *p*-value by univariate Cox regression adjusted to hours of life at hospital discharge.

b RR, relative risk; 95%CI, 95% confidence interval.

c 21 newborns without information on maternal ethnicity.

d RR of 37 week vs. ≥40 week.

e RR of AGA vs. small for age.

## Discussion

Risk factors for excessive weight loss in full-term infants that were exclusively breastfed and were born in a Baby-Friendly Hospital were cesarean section and older maternal age. At the multiple regression model, cesarean delivery remained the only independent factor for weight loss>8%.

Considering the policies of the Baby-Friendly Hospital, the mean loss observed in the total sample was similar to that in other studies. Thus, the mean weight loss of 6.4% is compatible with descriptions of 5.7% to 6.6%±2% in a systematic review of 11 very heterogeneous studies, with exclusively breastfed newborns and regardless of the type of delivery.^2^^,^ 14 As for the frequency of weight loss>8% in this study, it was a significant one (25.8% of the newborns). Different definitions of excessive weight loss have been used by authors, namely>7;>8 and>10%.^19^ The 8% cut-off is not very frequently used.^19^ We found one reference^14^ that described values of 7.4% of newborns with losses>8% on exclusive breastfeeding, not breastfeeding and formula. We chose the 8% threshold for excessive weight loss considering the need for breastfeeding supplementation in these infants after hospital discharge, as shown in step 10 of the Baby-Friendly Hospital.^8^^,^ 10

There is no consensus whether a weight loss>7% can indicate breastfeeding problems^7^^,^ 8 and, on the other hand, the loss of 8-10% can be considered physiological if there are no abnormalities at the physical examination. It indicates, however, the need for greater breastfeeding support.^8^^,^ 19

For losses>10%, the value obtained in this study is close to the lower limit of the wide range of variation found in other publications.^4^^,^ 5 ^,^ 11 ^,^ 15 ^,^ 20 ^-^ 22 Thus, 2.4^5^ to 25%^21^ of newborns lose more than 10% of weight according to studies with different types of feeding. The absence of newborns with weight loss>10% has also been described.^3^^,^ 14 We believe the percentage of 4.8% obtained in this study can be attributed to the implementation of all steps of the Baby-Friendly Hospital Initiative in the service.

Among the assessed risk factors, older maternal age was identified as a predictor of weight loss, probably because, with older age, there is an increase in maternal morbidity due to hypertension and diabetes, which develops into the risk of delivery by cesarean section.^23^

At the bivariate analysis between gestational age and weight loss and between age and the cesarean rate, a significant difference was identified only for the second: the newborns with 37 and 40 weeks of gestational age showed the highest frequencies of cesarean delivery. In turn, the multivariate regression analysis, which suppressed the confounding effects, did not show gestational age as a risk factor, which would be expected, as, in clinical practice, the 37-week newborns are the ones that show greater propensity to breastfeeding difficulties.^24^

The best-known mechanism that explains the association between cesarean delivery and greater weight loss of the newborn and breastfeeding problems is the delay in lactogenesis II, defined as copious milk production that starts on the 2nd/3rd day after birth.^11^^,^ 25 There are also known newborn positioning impediments in a cesarean delivery to meet step 4 of the Baby-Friendly Hospital, with the first feeding occurring within half to one hour of life, which might contribute to a delayed lactogenesis.^26^

More recently, studies have identified that another mechanism that would also be present is the excess of fluids administered to the mother during labor.^22^^,^ 27 The infusion of 1.200mL to more than 2.500mL of fluids to the mother, at a cesarean section or vaginal delivery with analgesia,^19^ determines hypervolemia in newborns and increases diuresis on the first day of life.^2^^,^ 27 Much of the weight loss of these newborns is observed on the first day.^2^^,^ 19 In a review of the subject^19^ evidence was found in eight studies that associated weight loss with excess fluids offered to the mother in exclusively breastfed newborns. On the other hand, a randomized clinical trial found no association between lower weight loss and fluid infusion restriction in mothers.^28^

This study constitutes a secondary data analysis of a prior study, of which information about weight gain was prospectively collected. Thus, the evaluation of other variables associated with weight loss and breastfeeding difficulties^13^^,^ 20 was not performed, which can be seen as a study limitation. Also, the longer duration of hospital length of stay after a cesarean delivery when compared to vaginal delivery would predispose to the observation of weight loss nadir in this first group.^21^ However, this effect was suppressed by adjusting the regression analysis for the length of stay in hours.

The weight at hospital discharge was the predictor of the degree of anxiety and concern about the milk production volume.^20^ Thus, if it is possible to know and minimize the factors associated with weight loss, perhaps it may also be possible to reduce maternal anxiety and contribute to higher success rates of exclusive breastfeeding.^20^ It is concluded that reducing the number of cesarean sections could minimize excessive neonatal weight loss, and consequently, minimize the indication of supplementation with formula on the first days of life.
